# *BRCA* genetic testing and treatment patterns for patients with HER2-negative early-stage breast cancer in the US community setting

**DOI:** 10.3389/fonc.2026.1730548

**Published:** 2026-02-27

**Authors:** Kathryn E. Mishkin, Yezhou Sun, Ke Meng, Xiaoqing Xu, Qixin Li, Yu-Han Kao, Jagadeswara Rao Earla, Kim M. Hirshfield, Jaime A. Mejia

**Affiliations:** 1 Value & Implementation Outcomes Research, Oncology, Merck & Co., Inc., Rahway, NJ, United States; 2 Biostatistics and Research Decision Sciences (BARDS), Merck & Co., Inc., Rahway, NJ, United States; 3 Oncology Outcomes Research, AstraZeneca, Gaithersburg, MD, United States; 4 MRL, Merck & Co., Inc., Rahway, NJ, United States

**Keywords:** adjuvant therapy, biomarker testing, *BRCA*, breast cancer, endocrine therapy, real-world study, recurrence, targeted therapy

## Abstract

**Background:**

Adjuvant olaparib has been approved in the US since 2022 for patients with high-risk HER2-negative early-stage breast cancer (eBC) with germline mutation in *BRCA1* or *BRCA2* (g*BRCA*m), and contemporary real-world data are needed regarding *BRCA*m testing patterns, *BRCA*m prevalence, and therapy selection for HER2-negative eBC in the US.

**Methods:**

This retrospective cohort study used a longitudinal, real-world dataset from US community healthcare systems to describe characteristics and *BRCA*m testing of adults with initial diagnosis from 1-Jan-2022 to 22-Jan-2024 of clinical stage I-III HER2-negative BC. Patients with unknown hormone receptor (HR) status, enrolled in a clinical trial, or with lobular carcinoma *in situ* or other primary cancer were excluded. Subsequent therapy was described by *BRCA*m status.

**Results:**

Among 3741 patients with HER2-negative eBC, 51% and 56% were *BRCA*m tested in 2022 and 2023, respectively, >99% for germline *BRCA*m, and including 70% of 509 patients with triple-negative breast cancer (TNBC) and 50% of 3232 patients with HR+/HER2-negative eBC. Of 1985 patients tested before a metastatic diagnosis, testing was conducted for 2% before initial diagnosis, for 77% at/before surgery, and for 21% after definitive surgery; 96 (5%) had *BRCA*-mutated eBC, including 51 of 1630 (3%) with HR+/HER2-negative eBC and 45 of 355 (13%) with TNBC. With median follow-up of 20.2 months, 1922 patients (97%) underwent surgery, with greater proportion of patients with *BRCA*-mutated eBC undergoing mastectomy (83% vs. 32% with no *BRCA*m). Most patients (>90%) received systemic therapy: 8% neoadjuvant-only, 65% adjuvant-only, and 18% both. Of 49 patients with *BRCA*-mutated HR+ eBC who had definitive surgery, 36 (73%) received adjuvant therapy, most commonly endocrine therapy-only (20/36, 59%). Of 44 patients with *BRCA*-mutated TNBC, 20 (45%) received adjuvant therapy, most commonly immunotherapy (11/20, 55%). Eighteen patients, all *BRCA*m tested, received adjuvant olaparib as monotherapy (6 patients) or in a combination regimen (12 patients).

**Conclusions:**

*BRCA*m testing rates in the US remain suboptimal relative to practice guideline recommendations and availability of adjuvant targeted therapy for HER2-negative eBC. Particular areas of unmet need include *BRCA*m testing for HR+/HER2-negative eBC, in addition to TNBC, and improving the timeline of *BRCA*m testing by implementing genetic testing before surgery to inform treatment decisions.

## 1 Introduction

Breast cancer is the most common cancer globally and the second most common cause of cancer deaths among women in the United States (US), with an estimated 317,000 new cases and 42,000 breast cancer-related deaths projected for the US in 2025 (1–4). The prognosis and selection of therapy for breast cancer is guided by clinical presentation, including patient age, tumor stage, and pathological features such as tumor subtype defined according to human epidermal growth factor receptor 2 (HER2) and estrogen and progesterone receptor (ER and PR) status. Approximately 70% of tumors are hormone receptor-positive (HR+) and HER2-negative, while triple-negative breast cancer (TNBC), defined as testing negative for all three receptors (ER, PR, and HER2), accounts for approximately 10% to 15% of all breast cancers and is more common among African American women and those with a *BRCA1* mutation (3–5).

The presence of a pathogenic or likely pathogenic variant in the *BRCA1* and/or *BRCA2* gene markedly increases the risk of breast cancer occurrence and its recurrence after standard therapy (6, 7). For patients with early-stage breast cancer, identification of a *BRCA* mutation (*BRCA*m) is an important consideration in therapeutic decision-making, both regarding surgical approach as well as choice of systemic therapy (8–11). The presence of a germline *BRCA*m is predictive of clinical response to specific therapies, particularly to poly(ADP-ribose) polymerase (PARP) inhibitors, such as olaparib (12, 13). Adjuvant olaparib received regulatory approval in the US in March 2022, based on results of the phase 3 OlympiA clinical trial (NCT02032823) demonstrating significant overall survival (OS) benefit of olaparib vs placebo, for treating adult patients with germline *BRCA*-mutated early-stage, high-risk HER2-negative breast cancer who had received definitive local therapy and neoadjuvant or adjuvant chemotherapy and who were selected for therapy based on an approved companion diagnostic test (14–16); and sustained benefit of the 1-year olaparib regimen was demonstrated at median 6-year follow-up (17). This followed the approval in January of 2018 of olaparib for patients with metastatic germline *BRCA*-mutated HER2-negative breast cancer who had received prior chemotherapy in the neoadjuvant, adjuvant, or metastatic setting (18).

Current US consensus guidelines for *BRCA*m testing differ somewhat in details, but all recommend genetic testing that includes both *BRCA1* and *BRCA2* for all patients with TNBC and male breast cancer and for patients with a personal history of breast cancer, with a pertinent family history, or with high-risk, HER2-negative breast cancer (to aid in adjuvant treatment decisions with olaparib) (19–21). Consensus guidelines of the American Society of Breast Cancer Surgeons have recommended genetic testing for all patients with breast cancer since 2019, to include at least *BRCA1*, *BRCA2*, and *PALB2* (19), while more recent joint guidelines from the American Society of Clinical Oncology (ASCO) and the Society of Surgical Oncology (SSO) recommend *BRCA*m testing for all patients ≤65 years with newly diagnosed stage I-III breast cancer and those >65 years old with TNBC or high-risk early-stage disease (20).

Reported *BRCA*m testing rates in the US for patients with breast cancer have been slowly increasing in the last decade (22–24). However, testing rates were only 34% among women and 61% among men with breast cancer diagnosis in 2019 and followed to March 2021 (24). Information about current real-world *BRCA* genetic testing patterns, *BRCA*m prevalence, and therapy selection for HER2-negative early-stage breast cancer in the US would aid in identifying areas of unmet need for this patient population. Our objectives were to describe *BRCA*m testing patterns and results for patients with HER2-negative early-stage breast cancer treated in US community settings and, for patients tested for *BRCA*m, to describe their characteristics and therapy, including adjuvant olaparib use.

## 2 Materials and methods

We followed the STROBE (Strengthening the Reporting of Observational Studies in Epidemiology) guideline in reporting this retrospective cohort study (25). The study protocol was approved by the Advarra Institutional Review Board, with a waiver of informed consent for the secondary analysis of existing data (26).

### 2.1 Data source

This study used the Syapse Learning Health Network (LHN), a dataset capturing the continuum of care for patients treated at US community practices, where >80% of patients with cancer in the US are treated (27), as distinct from academic medical centers or National Cancer Institute-Designated Cancer Centers. The dataset contains longitudinal medical data, including electronic health record data, laboratory and imaging results, and surgical and treatment records, from patients with cancer treated in community-based health care settings across 25 states. Dataset curation for this study included manual abstraction of key study variables and confirmation of two or more recorded distinct clinical encounters after a first breast cancer diagnosis for included patients. At the time of the study, the LHN included ~1350 participating oncologists and 1.6 million patients with cancer from five not-for-profit health systems. Syapse Holdings Inc. was acquired by N-Power Medicine on December 30, 2024 (27).

### 2.2 Patients

Eligible patients for the study were adults (≥18 years old) with an initial diagnosis of stage I, II, or III HER2-negative breast cancer, with known HR status, recorded from January 1, 2022, to January 22, 2024, thus recorded from approximately 2½ months before the adjuvant olaparib approval on March 11, 2022. Negative status for HER2 was defined as immunohistochemistry (IHC) 0, 1+, or 2+/*in situ* hybridization (ISH)-negative; in addition, we excluded patients who were prescribed any HER2-targeted therapy. Also excluded were patients enrolled in a clinical trial or with lobular carcinoma *in situ* (LCIS), pleomorphic LCIS, or lymphoma, in addition to those with other primary cancer before the breast cancer diagnosis (or with undocumented date), except for non-melanoma skin cancer. Data cutoff was on February 4, 2025.

### 2.3 Assessments and analyses

We evaluated whether patients had received *BRCA*m testing (germline or somatic) and, if yes, whether testing had been performed before development of metastatic breast cancer, including the frequency overall, by diagnosis year (2022, 2023, 2024), by patient demographics, by stage, and by HR status (HR+ or TNBC). Subsequent descriptive analyses focused on patients with a *BRCA*m test before metastasis, our main patient population of interest. For these patients, we examined the timeline of *BRCA*m testing relative to the initial diagnosis and surgery and summarized *BRCA*m status (mutation, no mutation, or unknown) overall and by HR status. We relied on sample source information (germline/somatic/unknown) provided in the dataset. Testing for *BRCA*m was captured from patients’ electronic health records; physician notes were not considered. The presence of a *BRCA1* or *BRCA2* mutation was defined as an explicit notation of a pathogenic or likely pathogenic variant or as a copy number variation (CNV) loss. Negative *BRCA*m status was inferred from results of test panels that included *BRCA*m testing and results indicated the genes were “not mutated” but did not provide an explicit test result.

Patterns of surgery, radiation therapy, and systemic anticancer therapy, including neoadjuvant and adjuvant therapy and olaparib use, were then summarized by *BRCA*m status, overall and according to HR status (HR+ or TNBC). Patients were followed from diagnosis to the earliest of other cancer, recurrence, death, or the last data abstraction.

No *a priori* hypothesis tests were planned; therefore, power and sample sizes were not estimated for formal hypothesis testing. Analyses were conducted using R version 4.3.3 (R Project for Statistical Computing) (28).

## 3 Results

### 3.1 Patient selection and *BRCA* mutation testing

**Figure 1:**
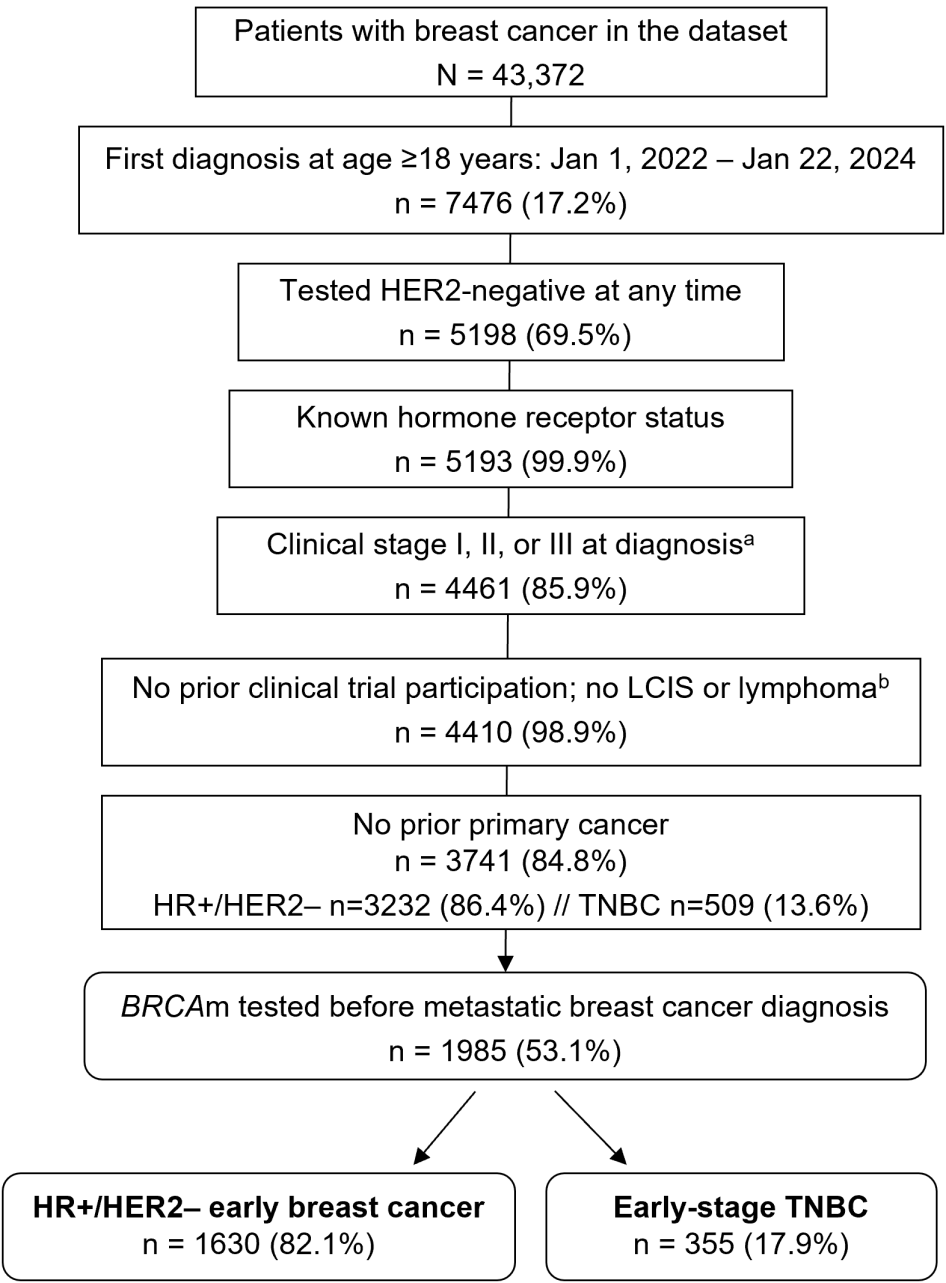
Selection of eligible patients. ^a^Excluding stages 0 or IV breast cancer and unknown stage. ^b^No patients were excluded for LCIS or lymphoma after those in a clinical trial were excluded. Percentages refer to the prior step in the flow diagram. *BRCA*m, *BRCA* mutation; HER2–, human epidermal growth factor receptor 2-negative; HR, hormone receptor; LCIS, lobular carcinoma *in situ*; TNBC, triple-negative breast cancer.

Of 43,372 patients in the LHN dataset with a breast cancer diagnosis, 7476 patients (17%) had an initial diagnosis from January 1, 2022, to January 22, 2024, and 5193/7476 (69%) had HER2-negative breast cancer with known HR status (**Figure 1**). After excluding 1452 patients with stage 0 or IV (or unknown stage), in a clinical trial, or with other primary cancer, the remaining 3741 patients included 3232 (86%) with HR+/HER2-negative breast cancer and 509 (14%) with TNBC, among whom 2000 patients were tested for *BRCA*m. Most were tested before a diagnosis of metastatic disease, namely, 1985 of 3741 patients (53%), our patient population of interest, including 1630 patients (82%) with HR+/HER2-negative breast cancer and 355 (18%) with TNBC (**Figure 1**).

The number of patients tested for *BRCA*m before a diagnosis of metastatic disease is summarized in **Table 1** for the 3741 patients with HER2-negative early-stage breast cancer. Testing was performed for 50% of patients with HR+ breast cancer and 70% of those with TNBC, with the greatest percentages tested among those <50 years old or with stage III disease (**Table 1**). A total of 51% of patients were *BRCA*m tested in 2022 and 56% in 2023; and testing for the three largest race categories (White, Black/African American, and Asian) ranged from 50% to 59%.

**Table 1:** Patterns of *BRCA* testing among patients with stage I, II, or III HER2-negative breast cance before a metastatic diagnosis.

Variable	N	*BRCA*m test before metastasis, n (%)
All eligible patients	3741	1985 (53.1)
Age		
≤50 years old	771	610 (79.1)
51–65 years old	1344	738 (54.9)
>65 years old	1626	637 (39.2)
Year of initial breast cancer diagnosis		
2022	2153	1100 (51.1)
2023	1575	878 (55.7)
2024	13	7 (53.8)
Hormone receptor status		
HR-positive	3232	1630 (50.4)
TNBC	509	355 (69.7)
Stage		
I	2854	1447 (50.7)
II	608	347 (57.1)
III	279	191 (68.5)
Race		
White	2894	1546 (53.4)
Black/African American	577	289 (50.1)
Asian	197	116 (58.9)
American Indian/Alaska Native	17	6 (35.3)
Native Hawaiian/Pacific Islander	9	5 (55.6)
Other	15	5 (33.3)
Race not documented	32	18 (56.2)
Ethnicity		
Hispanic/Latino	346	182 (52.6)
Non-Hispanic/non-Latino	3346	1777 (53.1)
Unknown	49	26 (53.1)

Percentages represent row percentages.

HR, hormone receptor; TNBC, triple-negative breast cancer.

Of the 1914 patients with known timing of the *BRCA*m test before metastasis, 45 patients (2%) were tested before the initial diagnosis, plus 1467 (77%) at or before surgery and 402 (21%) after definitive surgery and before a metastatic breast cancer diagnosis. Among 1969 patients with known genomic source, 1962 (>99%) were tested for g*BRCA*m and 7 (<1%) for somatic *BRCA*m.

### 3.2 Patient characteristics and results of *BRCA* mutation testing

The median age of all 1985 patients was 59 years (range, 23–89), including 31%, 37%, and 32% aged ≤50, 51–65, and >65 years old, respectively. All but 19 patients (1%) were women, and of the 1966 women, approximately two-thirds were postmenopausal (**Table 2**). The majority of patients were White (82%) and 12% were Black or African American in the HR+ cohort, while the percentages were 65% and 27%, respectively, in the TNBC cohort.

**Table 2:** Characteristics of patients at diagnosis of HER2-negative early breast cancer that was *BRCA* mutation tested before a metastatic breast cancer diagnosis.

Characteristic	All patients (N = 1985)	Hormone receptor status	
		HR+ (n = 1630)	TNBC (n = 355)
Age, median (range), y	59 (23-89)	59 (23-89)	58 (25-89)
≤50 years old	610 (30.7)	507 (31.1)	103 (29.0)
51–65 years old	738 (37.2)	589 (36.1)	149 (42.0)
≥65 years old	637 (32.1)	534 (32.8)	103 (29.0)
Sex			
Female	1966 (99.0)	1611 (98.8)	355 (100)
Male	19 (1.0)	19 (1.2)	0
Menopausal status (women only)^a^			
Postmenopausal	1301 (67.3)	1059 (66.6)	242 (70.6)
Perimenopausal	76 (3.9)	63 (4.0)	13 (3.8)
Premenopausal	556 (28.8)	468 (29.4)	88 (25.7)
Not documented	33	21	12
Race^a^			
White	1546 (78.6)	1317 (81.5)	229 (65.1)
Black/African American	289 (14.7)	193 (12.0)	96 (27.3)
Asian	116 (5.9)	92 (5.7)	24 (6.8)
Other^b^	16 (0.8)	13 (0.8)	3 (0.9)
Not documented	18	15	3
Region of the US			
Midwest	1597 (80.5)	1294 (79.4)	303 (85.4)
Northeast	195 (9.8)	169 (10.4)	26 (7.3)
South	193 (9.7)	167 (10.2)	26 (7.3)
Insurance			
Private insurance	1031 (51.9)	850 (52.1)	181 (51.0)
Medicare	690 (34.8)	581 (35.6)	109 (30.7)
Medicaid	170 (8.6)	127 (7.8)	43 (12.1)
Other	77 (3.9)	62 (3.8)	15 (4.2)
Not insured	17 (0.9)	10 (0.6)	7 (2.0)
ECOG performance status^a^			
0 or 1	870 (96.9)	651 (97.0)	219 (96.5)
≥2	28 (3.1)	20 (3.0)	8 (3.5)
Not documented	1087	959	128
Tumor histologic type			
Ductal carcinoma	1569 (79.0)	1240 (76.1)	329 (92.7)
Lobular carcinoma	255 (12.8)	247 (15.2)	8 (2.3)
Other, including mixed	161 (8.1)	143 (8.8)	18 (5.1)
*BRCA* mutation status			
*BRCA* mutation	96 (4.9)	51 (3.1)	45 (12.7)
No mutation	1876 (95.1)	1573 (96.5)	303 (85.4)
Result not documented	13	6 (0.4)	7 (2.0)
Clinical tumor classification			
T0/Tis to T2	1815 (91.4)	1517 (93.1)	298 (83.9)
T3 to T4	170 (8.6)	113 (6.9)	57 (16.1)
Nodal involvement			
Positive	315 (15.9)	202 (12.4)	113 (31.8)
Negative	1670 (84.2)	1428 (87.6)	242 (68.2)
Overall disease stage			
Stage I	1447 (72.9)	1330 (81.6)	117 (33.0)
Stage II	347 (17.5)	216 (13.3)	131 (36.9)
Stage III	191 (9.6)	84 (5.2)	107 (30.1)
Tumor grade^a^			
Grade 1	425 (21.7)	425 (26.3)	0
Grade 2	944 (48.2)	892 (55.3)	52 (15.2)
Grade 3	588 (30.0)	297 (18.4)	291 (84.8)
Not documented	28	16	12
ER positivity			
Positive (≥11%)	1592 (80.2)	1592 (97.7)	0
Low positive (1-10%)	29 (1.5)	29 (1.8)	0
Negative (<1%)	364 (18.3)	9 (0.6)	355 (100)
PR status			
Positive (1-100%)	1420 (71.5)	1420 (87.1)	0
Negative (<1%)	565 (28.5)	210 (12.9)	355 (100)

Data are n (%) unless otherwise noted. Percentages may not total 100.0 because of rounding.

ECOG, Eastern Cooperative Oncology Group; ER, estrogen receptor; HR, hormone receptor; PR, progesterone receptor; TNBC, triple-negative breast cancer.

a Percentages for race, ECOG performance status, and tumor grade were calculated for patients with available data.

b Other race included American Indian, Alaska Native, Native Hawaiian, and Pacific Islander.

Ductal carcinoma was most common in both cohorts, including 76% and 93% in HR+ and TNBC cohorts, respectively (**Table 2**). Tumor characteristics differed between the two cohorts: at diagnosis, 7% and 16% of patients in HR+ and TNBC cohorts, respectively, had tumors assessed clinically as T3–T4; 12% and 32% had nodal involvement; 5% and 30% had stage III tumors; and 18% and 85%, respectively, had grade 3 disease.

The 96 patients with *BRCA*-mutated breast cancer included 51 of 1630 patients (3%) in the HR+ cohort and 45 of 355 (13%) in the TNBC cohort (**Table 2**). Almost two-thirds of the 96 patients (62%) with *BRCA*m were 50 years of age or younger. In the HR+ cohort 35 (69%), 7 (14%), and 9 patients (18%) were ≤50, 51–65, and >65 years old, respectively, and in the TNBC cohort, 24 (53%), 16 (36%) and 5 (11%) were ≤50, 51–65, and >65 years old, respectively.

### 3.3 Follow-up and therapy

The study follow-up time from breast cancer diagnosis to the earliest of other cancer diagnosis, recurrence, death, or the last data abstraction for individual patients was a median of 20.2 months (range, 0.3–33.3; interquartile range, 14.9–25.4 months). From surgery, the median follow-up time was 18.1 months for patients in the HR+ cohort and 14.4 months for those in the TNBC cohort.

#### 3.3.1 Surgery and radiation therapy

A total of 1922 of 1985 patients (97%) underwent surgery, among them 1246 (65%) who had breast-conserving surgery and 674 (35%) who had a mastectomy, the latter group including 77 of the 93 patients (83%) with *BRCA*-mutated breast cancer (**Table 3**, **Supplementary Tables 1**-**3**) and 112 of 175 patients (64%) with stage III disease who underwent surgery (**Supplementary Table 3**). Overall, 1371 of 1985 patients (69%) received radiation at the primary site, most of them in the adjuvant setting (1330, 97%; **Table 3**).

**Table 3:** Surgery, radiation therapy, and systemic therapy overall and by *BRCA* mutation status.

Treatment	All patients (N = 1985)	*BRCA* mutation status		
		*BRCA*m (n = 96)	Negative (n = 1876)	Unknown (n = 13)
Definitive surgery^a^	1922 (96.8)	93 (96.9)	1816 (96.8)	13 (100)
Breast-conserving	1246 (64.8)	16 (17.2)	1226 (67.5)	4 (30.8)
Mastectomy	674 (35.1)	77 (82.8)	588 (32.4)	9 (69.2)
Other	2 (0.1)	0	2 (0.1)	0
Radiation to primary site^a^	1371 (69.1)	44 (45.8)	1319 (70.3)	8 (61.5)
Neoadjuvant	30 (2.2)	0	30 (2.3)	0
Adjuvant	1330 (97.0)	44 (100)	1278 (96.9)	8 (100)
No surgery	11 (0.8)	0	11 (0.8)	0
Neoadjuvant/adjuvant therapy				
All HER2– eBC, N^b^	1922	93	1816	13
Neoadjuvant only	145 (7.5)	27 (29.0)	116 (6.4)	2 (15.4)
Adjuvant only	1244 (64.7)	23 (24.7)	1216 (67.0)	5 (38.5)
Neoadjuvant + adjuvant	348 (18.1)	33 (35.5)	310 (17.1)	5 (38.5)
No neoadjuvant or adjuvant^c^	185 (9.6)	10 (10.8)	174 (9.6)	1 (7.7)
HR+/HER2– cohort, N^b^	1583	49	1528	6
Neoadjuvant only	49 (3.1)	7 (14.3)	41 (2.7)	1 (16.7)
Adjuvant only	1179 (74.5)	20 (40.8)	1155 (75.6)	4 (66.7)
Neoadjuvant + adjuvant	196 (12.4)	16 (32.7)	179 (11.7)	1 (16.7)
No neoadjuvant or adjuvant^c^	159 (10.0)	6 (12.2)	153 (10.0)	0
TNBC cohort, N^b^	339	44	288	7
Neoadjuvant only	96 (28.3)	20 (45.5)	75 (26.0)	1 (14.3)
Adjuvant only	65 (19.2)	3 (6.8)	61 (21.2)	1 (14.3)
Neoadjuvant + adjuvant	152 (44.8)	17 (38.6)	131 (45.5)	4 (57.1)
No neoadjuvant or adjuvant^c^	26 (7.7)	4 (9.1)	21 (7.3)	1 (14.3)

All data are n (%) unless otherwise indicated. Percentages may not total 100.0 because of rounding.

eBC, early breast cancer; HER2–, HER2-negative; HR+, hormone receptor-positive; TNBC, triple-negative breast cancer.

a The patient percentages with definitive surgery and radiation are calculated as percentages of all patients, whereas the subcategories are percentages of those with definitive surgery or radiation therapy.

b Numbers (N) represent patients who underwent surgery.

c No neoadjuvant or adjuvant therapy was received, or either one was not documented.

#### 3.3.2 Systemic therapy

Most patients, 90% overall, received neoadjuvant, adjuvant, or both neoadjuvant and adjuvant systemic therapy (**Table 3**, **Supplementary Tables 1**-**3**). The most common regimens administered are summarized in **Table 4**. A total of 93 of 96 patients (97%) with *BRCA*-mutated early-stage breast cancer underwent surgery, and most (83/93, 89%) received systemic therapy regardless of setting (neoadjuvant or adjuvant therapy, or both). Of the 49 patients with *BRCA*-mutated HR+/HER2-negative breast cancer who had definitive surgery, 36 (73%) received adjuvant therapy, most commonly endocrine therapy only (20/36 patients, 59%; **Table 5**). Of the 44 patients with *BRCA*-mutated TNBC, 20 (45%) received adjuvant therapy, most commonly immunotherapy (11/20 patients, 55%).

**Table 4:** Neoadjuvant and adjuvant regimens administered to ≥10% of patients overall, and by *BRCA* mutation status.

Systemic therapy	All patients	*BRCA* mutation status		
		*BRCA*m	Negative	Unknown
All surgical patients, N	1922	93	1816	13
Neoadjuvant only	145 (7.5)	27 (29.0)	116 (6.4)	2 (15.4)
Chemo + IO	70 (48.3)	16 (59.3)	54 (46.6)	0
Chemo	42 (29.0)	8 (29.6)	32 (27.6)	2 (100)
ET	29 (20.0)	2 (7.4)	27 (23.3)	0
Adjuvant only	1244 (64.7)	23 (24.7)	1216 (67.0)	5 (38.5)
ET	947 (76.1)	14 (60.9)	929 (76.4)	4 (80.0)
Chemo + ET	173 (13.9)	4 (17.4)	169 (13.9)	0
Both: Neoadjuvant // Adjuvant	348 (18.1)	33 (35.5)	310 (17.1)	5 (38.5)
Chemo + IO // IO	95 (27.3)	10 (30.3)	84 (27.1)	1 (20.0)
Chemo // ET + TT	48 (13.8)	5 (15.2)	43 (13.9)	0
Chemo // ET	46 (13.2)	5 (15.2)	40 (12.9)	1 (20.0)
HR+/HER2– cohort, N	1583	49	1528	6
Neoadjuvant only	49 (3.1)	7 (14.3)	41 (2.7)	1 (16.7)
ET	29 (59.2)	2 (28.6)	27 (65.9)	0
Chemo	10 (20.4)	2 (28.6)	7 (17.1)	1 (100)
Chemo + IO	8 (16.3)	3 (42.9)	5 (12.2)	0
Adjuvant only	1179 (74.5)	20 (40.8)	1155 (75.6)	4 (66.7)
ET	947 (80.3)	14 (70.0)	929 (80.4)	4 (100)
Chemo + ET	171 (14.5)	3 (15.0)	168 (14.5)	0
Both: Neoadjuvant // Adjuvant	196 (12.4)	16 (32.7)	179 (11.7)	1 (16.7)
Chemo // ET + TT	48 (24.5)	5 (31.2)	43 (24.0)	0
Chemo // ET	46 (23.5)	5 (31.2)	40 (22.3)	1 (100)
ET // ET	23 (11.7)	0	23 (12.8)	0
TNBC cohort, N	339	44	288	7
Neoadjuvant only	96 (28.3)	20 (45.5)	75 (26.0)	1 (14.3)
Chemo + IO	62 (64.6)	13 (65.0)	49 (65.3)	0
Chemo	32 (33.3)	6 (30.0)	25 (33.3)	1 (100)
Adjuvant only	65 (19.2)	3 (6.8)	61 (21.2)	1 (14.3)
Chemo	58 (89.2)	1 (33.3)	57 (93.4)	0
Both: Neoadjuvant // Adjuvant	152 (44.8)	17 (38.6)	131 (45.5)	4 (57.1)
Chemo + IO // IO	85 (55.9)	10 (58.8)	74 (56.5)	1 (25.0)
Chemo + IO // Chemo + IO	18 (11.8)	0	18 (13.7)	0
Chemo + IO // Chemo	15 (9.9)	0	15 (11.5)	0
Chemo // Chemo	15 (9.9)	0	15 (11.5)	0

All data are n (%) unless otherwise indicated. Percentages may not total 100.0 because of rounding and because regimens administered to <10% of patients overall are not included.

Chemo, chemotherapy; ET, endocrine therapy; HER2–, HER2-negative; HR+, hormone receptor-positive; IO, immunotherapy; TNBC, triple-negative breast cancer; TT, targeted therapy.

**Table 5:** Adjuvant regimens administered to 93 patients with *BRCA*-mutated breast cancer, by hormone receptor status.

HR+/HER2-negative (N = 49)	n (%)	TNBC (N = 44)	n (%)
ET only	20 (40.8)	IO only	11 (25.0)
ET + olaparib	4 (8.2)	IO + olaparib	3 (6.8)
ET + CDK4/6i	4 (8.2)	Olaparib	2 (4.5)
Chemo + ET	4 (8.2)	Chemo + IO + olaparib	2 (4.5)
ET + olaparib + CDK4/6i	1 (2.0	Chemo	1 (2.3)
Chemo	1 (2.0)	Chemo + ET	1 (2.3)
Chemo + olaparib	1 (2.0)	--	--
Chemo + ET + IO + olaparib	1 (2.0)	--	--
No adjuvant therapy	13 (26.5)	No adjuvant therapy	24 (54.5)

Percentages may not total 100.0 because of rounding.

CDK4/6i, cyclin-dependent kinase 4/6 inhibitor; Chemo, chemotherapy; ET, endocrine therapy; HR+, hormone receptor-positive; IO, immunotherapy; TNBC, triple-negative breast cancer.

A total of 18 patients received adjuvant olaparib, either as monotherapy (6 patients) or as part of a combination regimen (12 patients). All 18 had been *BRCA*m tested, among them 14 with *BRCA*-mutated breast cancer (adjuvant regimens summarized in **Table 5**), 3 with undocumented test results (*BRCA*m status unknown), and 1 with negative results. Olaparib therapy was ongoing for 14 of the 18 patients at data cutoff.

## 4 Discussion

In this retrospective study of patients with HER2-negative early-stage breast cancer, we found that just over half of 3741 patients treated in US community settings were tested for *BRCA*m in 2022 and 2023 (51% and 56%, respectively), with greater frequency overall among patients with TNBC (70%) than with HR+/HER2-negative breast cancer (50%). Moreover, 79% of patients ≤50 years old were tested, as compared with 39% of those >65 years old. The percentages of patients *BRCA*m tested were in the 50% to 59% range for White, Black/African American, and Asian individuals, the three largest racial groups, as well as for Hispanic/Latino and non-Hispanic/non-Latino individuals. Over 99% of the 1969 patients with a known genomic source were tested for g*BRCA*m, with a small minority (n=7) tested for somatic *BRCA*m. Positive test results for *BRCA*m were recorded for 3% of patients with HR+/HER2-negative disease and for 13% with TNBC. Of the patients with *BRCA*-mutated breast cancer, the majority (93 of 96) underwent definitive surgery and received neoadjuvant or adjuvant therapy, or both, including 14 (15%) who received adjuvant olaparib.

Our findings suggest that *BRCA*m testing rates continue to slowly increase in the US. In another study of adult women of all ages (≥20 years old) using data from the Surveillance, Epidemiology, and End Results (SEER) registries in California and Georgia, testing rates rose from 22% to 34% for women with breast cancer diagnosis in 2013 to 2019 and followed through the first quarter of 2021 (24) (vs. 51%–56% in 2022–2023 in our study). An earlier study using the same SEER registries for women with breast cancer diagnosed in 2013–2014 found that genetic testing rates were greater for women with TNBC (40%) than for those with HR+/HER2-negative subtype (23%) (29), a similar pattern to our more recent findings (70% vs. 50%, respectively). In a prior study of 28,655 older women, ages ≥66 years, with diagnosis in 2010–2017 of HER2-negative early-stage breast cancer, and who underwent primary surgery, 7578 (26%) were *BRCA*m tested (30); this percentage was 39% among women >65 years old in our study.

Different from the SEER registry study, which reported lower testing rates among Asian, Black, and Hispanic patients compared with White patients (24), we found that the percentages of patients tested were relatively similar across racial groups, although with the caveat that there are potential differences in patient characteristics and insurance coverage between our largely Midwestern, community-based patient population and the SEER registries in California and Georgia. We observed, as expected, that tumor characteristics at diagnosis indicated more high-risk disease among patients with TNBC than those with HR+ tumors, with greater percentages of TNBC at stage III, with nodal involvement, with grade 3 disease, and with *BRCA*m. Moreover, as generally accepted (3–5), Black/African American patients were proportionately over-represented in the TNBC cohort (comprising 33% of all 289 Black/African American patients) as compared with White patients (15% of all 1546 White patients).

The March 2022 approval of adjuvant olaparib for high-risk early-stage *BRCA*-mutated breast cancer provided the first available adjuvant targeted therapy for the patients in this study (with initial diagnosis from January 1, 2022, to January 22, 2024) and theoretically could have provided motivation for increased *BRCA*m testing. Nonetheless, 30% of patients with TNBC did not have a *BRCA*m test, despite practice guideline recommendations (19). Of the 96 patients with *BRCA*m in this study, 39% were >50 years old and 15% were >65 years old, highlighting the importance of *BRCA*m testing for patients of all ages with stages I-III HER2-negative breast cancer. Moreover, we observed that approximately one-fifth of patients (21%) had *BRCA*m testing after definitive surgery, which meant that *BRCA*m test results were available too late to factor into surgical decision-making and possibly too late to influence adjuvant therapy decisions. Prior research has suggested that patients are more likely to agree to receive *BRCA*m testing based on a recommendation by their health care provider and as an opportunity to be fully informed about their breast cancer (31). Providers are more likely to recommend *BRCA*m testing for patients with a family history, at younger ages, and with TNBC; patient refusal and insurance issues were reported as barriers to *BRCA*m testing (32). In their recent review of genetic testing for *BRCA*m breast cancer, Arun and coworkers propose several strategies to improve testing rates, including population-based screening of individuals at high risk of *BRCA*m, free genetic counseling, and provision of training and education for health care providers, together with increasing awareness for patients (33). Additional studies around factors to facilitate *BRCA*m testing may be helpful to increase testing rates, to identify patients with *BRCA*-mutated breast cancer who could benefit from targeted treatments, and to better inform surgical decisions.

A total of 18 patients with HER2-negative early-stage breast cancer in this study received adjuvant olaparib, and all 18 patients had received a *BRCA*m test, as recommended. Similarly, a recent US retrospective study found that, of 134 olaparib users with HER2-negative early-stage breast cancer, all had received at least one *BRCA*m test (germline and/or somatic) (34). Further study of a more contemporary cohort will extend our understanding of *BRCA*m genetic testing patterns and olaparib use in this patient population.

This study used a large database with follow-up until early 2025 to study *BRCA*m testing among almost 3800 patients with HER2-negative early-stage breast cancer treated at US community practices. The database included detailed clinical information, with minimal missing data regarding tumor characteristics at diagnosis, menopausal status, and insurance status, although ECOG performance status was not documented for approximately half of patients. However, the fact that we required documented HER2 and HR status may have resulted in selection bias favoring patients who were more likely to have comprehensive diagnostic testing, including *BRCA*m tests, and those with complete documentation in their medical records. Moreover, the generalizability of our findings may be limited beyond the included health systems, most in the Midwest, or for patients treated at academic centers. In addition, as for all database studies, we cannot rule out the limitation of possible errors in data recording. Finally, this descriptive study enrolled patients with early-stage breast cancer, for whom lengthy follow-up is needed to evaluate recurrence and survival outcomes. Future long-term studies are needed to evaluate the outcomes of different surgical approaches and neoadjuvant and adjuvant therapy selection for patients with HER2-negative early-stage breast cancer according to *BRCA*m status and by HR status.

## 5 Conclusions

Testing rates for *BRCA*m in the US remain suboptimal relative to practice guideline recommendations and the availability of adjuvant targeted therapy. Particular areas of unmet need include *BRCA*m testing for more patients with HER2-negative early-stage breast cancer, particularly those with HR+ disease and including patients >50 years of age. Moreover, improvements are needed in testing timelines by implementing genetic testing before surgery to guide surgical and systemic therapy decision-making. Future work will investigate use of olaparib for HER2-negative early-stage breast cancer in the US in subsequent years.

## Data Availability

The data analyzed in this study are subject to the following licenses/restrictions: We will adhere to the ethical obligations for responsible sharing of data. The data that support the findings of this study are not publicly available due to patient privacy and legal restrictions.. Requests to access these datasets should be directed to N-Power Medicine, support@npowermedicine.com.
