# Supplementary material for: BRCA genetic testing and treatment patterns for patients with HER2-negative early-stage breast cancer in the US community setting
Source: Front Oncol. 2026 Feb 27;16:1730548. doi: 10.3389/fonc.2026.1730548 (PMC12983088; doi:10.3389/fonc.2026.1730548)
Supplement: Supplementary file 1 [file Table1.docx]

Supplementary Material

# Supplementary Tables

**SUPPLEMENTARY TABLE 1** Surgery, radiation therapy, and systemic therapy overall and by *BRCA* mutation status: stage I HER2-negative early breast cancer.

| **Treatment** | **All patients**  **(N = 1447)** | ***BRCA* mutation status** | | |
| --- | --- | --- | --- | --- |
|  |  | ***BRCA*m**  **(n = 44)** | **No *BRCA*m**  **(n = 1396)** | **Unknown**  **(n = 7)** |
| Definitive surgery^a^ | 1419 (98.1) | 44 (100) | 1368 (98) | 7 (100) |
| Breast-conserving | 1033 (72.8) | 11 (25.0) | 1019 (74.5) | 3 (42.9) |
| Mastectomy | 385 (27.1) | 33 (75.0) | 348 (25.4) | 4 (57.1) |
| Other | 1 (<0.1) | 0 | 1 (<0.1) | 0 |
| Radiation to primary site^a^ | 1011 (69.9) | 14 (31.8) | 994 (71.2) | 3 (42.9) |
| Neoadjuvant | 29 (2.9) | 0 | 29 (2.9) | 0 |
| Adjuvant | 977 (96.6) | 14 (100) | 960 (96.6) | 3 (100) |
| No surgery | 5 (0.5) | 0 | 5 (0.5) | 0 |
| **Neoadjuvant/adjuvant therapy** |  |  |  |  |
| All HER2– eBC, N^b^ | 1419 | 44 | 1368 | 7 |
| Neoadjuvant only | 54 (3.8) | 9 (20.5) | 44 (3.2) | 1 (14.3) |
| Adjuvant only | 1139 (80.3) | 21 (47.7) | 1114 (81.4) | 4 (57.1) |
| Neoadjuvant + adjuvant | 61 (4.3) | 4 (9.1) | 56 (4.1) | 1 (14.3) |
| No neoadjuvant or adjuvant^c^ | 165 (11.6) | 10 (22.7) | 154 (11.3) | 1 (14.3) |
| HR+/HER2– cohort, N^b^ | 1303 | 30 | 1269 | 4 |
| Neoadjuvant only | 26 (2.0) | 2 (6.7) | 24 (1.9) | 0 |
| Adjuvant only | 1083 (83.1) | 18 (60.0) | 1061 (83.6) | 4 (100) |
| Neoadjuvant + adjuvant | 45 (3.5) | 4 (13.3) | 41 (3.2) | 0 |
| No neoadjuvant or adjuvant^c^ | 149 (11.4) | 6 (20.0) | 143 (11.3) | 0 |
| TNBC cohort, N^b^ | 116 | 14 | 99 | 3 |
| Neoadjuvant only | 28 (24.1) | 7 (50.0) | 20 (20.2) | 1 (33.3) |
| Adjuvant only | 56 (48.3) | 3 (21.4) | 53 (53.5) | 0 |
| Neoadjuvant + adjuvant | 16 (13.8) | 0 | 15 (15.2) | 1 (33.3) |
| No neoadjuvant or adjuvant^c^ | 16 (13.8) | 4 (28.6) | 11 (11.1) | 1 (33.3) |

All data are n (%) unless otherwise indicated. Percentages may not total 100.0 because of rounding.

eBC, early breast cancer; TNBC, triple-negative breast cancer.

^a^The patient percentages with definitive surgery and radiation are calculated as percentages of all patients, whereas the subcategories are percentages of those with definitive surgery or radiation therapy.

^b^Numbers (N) represent patients who underwent surgery.

^c^No neoadjuvant or adjuvant therapy was received, or either one was not documented.

**SUPPLEMENTARY TABLE 2** Surgery, radiation therapy, and systemic therapy overall and by *BRCA* mutation status: stage II HER2-negative early breast cancer.

| **Treatment** | **All patients**  **(N = 347)** | ***BRCA* mutation status** | | |
| --- | --- | --- | --- | --- |
|  |  | ***BRCA*m**  **(n = 30)** | **No *BRCA*m**  **(n = 313)** | **Unknown**  **(n = 4)** |
| Definitive surgery^a^ | 328 (94.5) | 29 (96.7) | 295 (94.2) | 4 (100) |
| Breast-conserving | 151 (46.0) | 3 (10.3) | 147 (49.8) | 1 (25.0) |
| Mastectomy | 177 (54.0) | 26 (89.7) | 148 (50.2) | 3 (75.0) |
| Other | 0 | 0 | 0 | 0 |
| Radiation to primary site^a^ | 225 (64.8) | 14 (46.7) | 208 (66.5) | 3 (75.0) |
| Neoadjuvant | 0 | 0 | 0 | 0 |
| Adjuvant | 224 (99.6) | 14 (100) | 207 (99.5) | 3 (100) |
| No surgery | 1 (0.4) | 0 | 1 (0.5) | 0 |
| **Neoadjuvant/adjuvant therapy** |  |  |  |  |
| All HER2– eBC, N^b^ | 328 | 29 | 295 | 4 |
| Neoadjuvant only | 49 (14.9) | 8 (27.6) | 40 (13.6) | 1 (25.0) |
| Adjuvant only | 100 (30.5) | 2 (6.9) | 97 (32.9) | 1 (25.0) |
| Neoadjuvant + adjuvant | 167 (50.9) | 19 (65.5) | 146 (49.5) | 2 (50.0) |
| No neoadjuvant or adjuvant^c^ | 12 (3.7) | 0 | 12 (4.1) | 0 |
| HR+/HER2– cohort, N^b^ | 203 | 15 | 186 | 2 |
| Neoadjuvant only | 14 (6.9) | 4 (26.7) | 9 (4.8) | 1 (50) |
| Adjuvant only | 91 (44.8) | 2 (13.3) | 89 (47.8) | 0 (0) |
| Neoadjuvant + adjuvant | 92 (45.3) | 9 (60) | 82 (44.1) | 1 (50.0) |
| No neoadjuvant or adjuvant^c^ | 6 (3.0) | 0 | 6 (3.2) | 0 |
| TNBC cohort, N^b^ | 125 | 14 | 109 | 2 |
| Neoadjuvant only | 35 (28.0) | 4 (28.6) | 31 (28.4) | 0 |
| Adjuvant only | 9 (7.2) | 0 | 8 (7.3) | 1 (50.0) |
| Neoadjuvant + adjuvant | 75 (60.0) | 10 (71.4) | 64 (58.7) | 1 (50.0) |
| No neoadjuvant or adjuvant^c^ | 6 (4.8) | 0 | 6 (5.5) | 0 |

All data are n (%) unless otherwise indicated. Percentages may not total 100.0 because of rounding.

eBC, early breast cancer; TNBC, triple-negative breast cancer.

^a^The patient percentages with definitive surgery and radiation are calculated as percentages of all patients, whereas the subcategories are percentages of those with definitive surgery or radiation therapy.

^b^Numbers (N) represent patients who underwent surgery.

^c^No neoadjuvant or adjuvant therapy was received, or either one was not documented.

**SUPPLEMENTARY TABLE 3** Surgery, radiation therapy, and systemic therapy overall and by *BRCA* mutation status: stage III HER2-negative early breast cancer.

| **Treatment** | **All patients**  **(N = 191)** | ***BRCA* mutation status** | | |
| --- | --- | --- | --- | --- |
|  |  | ***BRCA*m**  **(n = 22)** | **No *BRCA*m**  **(n = 167)** | **Unknown**  **(n = 2)** |
| Definitive surgery^a^ | 175 (91.6) | 20 (90.9) | 153 (91.6) | 2 (100) |
| Breast-conserving | 62 (35.4) | 2 (10.0) | 60 (39.2) | 0 |
| Mastectomy | 112 (64.0) | 18 (90.0) | 92 (60.1) | 2 (100) |
| Other | 1 (0.6) | 0 | 1 (0.7) | 0 |
| Radiation to primary site^a^ | 135 (70.7) | 16 (72.7) | 117 (70.1) | 2 (100) |
| Neoadjuvant | 1 (0.7) | 0 | 1 (0.9) | 0 |
| Adjuvant | 129 (95.6) | 16 (100) | 111 (94.9) | 2 (100) |
| No surgery | 5 (3.7) | 0 | 5 (4.3) | 0 |
| **Neoadjuvant/adjuvant therapy** |  |  |  |  |
| All HER2– eBC, N^b^ | 175 | 20 | 153 | 2 |
| Neoadjuvant only | 42 (24.0) | 10 (50.0) | 32 (20.9) | 0 |
| Adjuvant only | 5 (2.9) | 0 | 5 (3.3) | 0 |
| Neoadjuvant + adjuvant | 120 (68.6) | 10 (50.0) | 108 (70.6) | 2 (100) |
| No neoadjuvant or adjuvant^c^ | 8 (4.6) | 0 | 8 (5.2) | 0 |
| HR+/HER2– cohort, N^b^ | 77 (44.0) | 4 | 73 | 0 |
| Neoadjuvant only | 9 (11.7) | 1 (25.0) | 8 (11.0) | 0 |
| Adjuvant only | 5 (6.5) | 0 | 5 (6.8) | 0 |
| Neoadjuvant + adjuvant | 59 (76.6) | 3 (75.0) | 56 (76.7) | 0 |
| No neoadjuvant or adjuvant^c^ | 4 (5.2) | 0 | 4 (5.5) | 0 |
| TNBC cohort, N^b^ | 98 | 16 | 80 | 2 |
| Neoadjuvant only | 33 (33.7) | 9 (56.2) | 24 (30.0) | 0 |
| Adjuvant only | 0 | 0 | 0 | 0 |
| Neoadjuvant + adjuvant | 61 (62.2) | 7 (43.8) | 52 (65.0) | 2 (100) |
| No neoadjuvant or adjuvant^c^ | 4 (4.1) | 0 | 4 (5.0) | 0 |

All data are n (%) unless otherwise indicated. Percentages may not total 100.0 because of rounding.

eBC, early breast cancer; TNBC, triple-negative breast cancer.

^a^The patient percentages with definitive surgery and radiation are calculated as percentages of all patients, whereas the subcategories are percentages of those with definitive surgery or radiation therapy.

^b^Numbers (N) represent patients who underwent surgery.

^c^No neoadjuvant or adjuvant therapy was received, or either one was not documented.
